# Commentary on Lahiri et al. Weight and Body Mass Index Change After Switching to Integrase Inhibitors or Tenofovir Alafenamide Among Women Living with HIV

**DOI:** 10.33696/AIDS.3.016

**Published:** 2021

**Authors:** Francesca Macaluso, Deborah R. Gustafson

**Affiliations:** 1 Department of Medicine, State University of New York at Downstate Health Sciences University, Brooklyn NY, United States; 2 Department of Neurology, Section for NeuroEpidemiology, State University of New York Downstate Health Sciences University, Brooklyn, New York, USA

Among women living with HIV (WLWH), increases in body weight and body mass index (BMI, kg/m^2^) have been observed after switching to the antiretroviral therapies (ART) - Integrase Inhibitors (INSTI) and/or Tenofovir Alafenamide (TAF) [1,2]. This has broad implications for the HIV aging process and occurrence of later-life disease. While life expectancy for people living with HIV has increased, primarily due to highly-effective ART, the course of aging with chronic HIV remains relatively unknown. Particularly of interest are long-term ART consequences on individual physiological processes and end-organ systems. Increasing BMI leads to overweight and obesity, primarily due to excess adipose tissue accumulation. This is a sincere consequence of certain ART, mostly observed with use of the newer INSTI, specifically dolutegravir, compared to other INSTI [3–6]. While mechanisms whereby INSTI cause weight gain are not well understood, HIV infection has adverse metabolic and aging-related complications that are further complicated by the overweight and obesity-related side effects of ART in societies where obesity is a chronic disease [7], and overweight and obesity are pandemic [8]. Overweight and obesity have major medical, social, and economic repercussions [8]. Consequently, we can expect more ART-related cardiovascular and cerebrovascular sequelae, as well as polypharmacy to control both HIV infection and vascular risk. The paper by Lahiri, et al. [1] offers insights into the role of ART, specifically INSTI and TAF, on simple clinical measures of body weight and BMI.

Our group’s interest is the role of overweight and obesity on health of the aging brain, particularly in WLWH, and uninfected women [9–11]. Through decreases in cerebrospinal fluid HIV viral loads that reflect HIV in the central nervous system, ART has reduced the occurrence of severe HIV-Associated Neurocognitive Disorder (HAND) [12]. With extended survival however, one must consider the occurrence of late-onset cognitive impairments and dementias, including Alzheimer’s Disease and Related Dementias (ADRD), and Vascular Cognitive Impairments and Dementia (VCID) [13]. In addition, ART may induce other pathophysiological mechanisms leading to cognitive impairments and dementias *via* overweight and obesity and/or vascular and metabolic aberrations. Higher body weight, BMI, and central obesity among middle-aged, uninfected populations have been positively associated with subsequent late-onset cognitive impairments, ADRD and VCID [13–16]. Increased body weight and/or BMI among initially, non-obese INSTI and TAF users, are thus, particularly relevant when considering long-term cognitive health for people living with HIV infection. Whether WLWH will experience similar associations between overweight and obesity at middle-age and late-onset ADRD or VCID, and/or whether INSTI ART regimens further increase their risk, need to be monitored over time.

The paper by Lahiri, et al. [1] elicits several questions when considering overweight and obesity in relation to brain health. Over a follow-up period of 1–2 years, WLWH experienced body weight and BMI increases of 1–5 kg and 1–2 kg/m^2^, respectively. This is an alarming increase if continued over decades. These observed changes were dependent on 1) initial BMI, and 2) ART switch to INSTI only or INSTI+TAF. Questions related to this include: 1) Do women who switch to certain ART regimens continue to gain body weight and BMI over time? 2) Do women reach an inflection point and subsequently decline? 3) Do women plateau at a certain level of body weight and/ or BMI? Furthermore, is there an absolute or percent gain in body weight or BMI that increases risk of cognitive impairments and ADRD later in life, both independently and/or in combination with concomitant vascular risk factors? Another issue includes control of vascular risk factors such as hypertension, hyperlipidemia or type 2 diabetes. Are medical interventions for HIV and/or vascular conditions merely compressing middle-aged morbidity? In addition, as women appear to be more affected by adverse vascular and metabolic effects of ART, does sex or gender alone pose a risk for later-life ADRD as observed in some studies of aging [17,18]? Answers to these questions may inform uninfected populations, where data are sparse on this degree of iatrogenic body weight or BMI gain over a short time. However, some Type 2 diabetes medications such as insulin, and less commonly used sulfonylureas and thiazolidinediones, cause body weight gain, while others cause weight loss or are neutral [19]. Acetylcholinesterase inhibitors used for treatment of AD cause body weight loss during a life stage when weight loss is suboptimal for healthy survival [20].

The incidence and prevalence of cognitive impairments and ADRD, including VCID is expected to triple by 2050 [13]. The debilitating and resource-consuming sequelae of overweight and obesity affecting both brain and periphery, are thus worth investigating, particularly among cognitively-vulnerable groups, such as WLWH. Understanding the short-term adverse vascular and metabolic side effects of INSTI and TAF ART, such as body weight and BMI gain, warrants close, long-term follow up to determine the lifetime vascular and metabolic profiles of patients on these regimens, and potential impact on cognition, ADRD and VCID.

## References

[1] Lahiri CD, Xu Y, Wang K, Alvarez JA, Sheth AN, O’Halloran J, Weight and body mass index change after switching to integrase inhibitors or tenofovir alafenamide among women living with HIV. AIDS Research and Human Retroviruses. 2021 1 12. 10.1089/aid.2020.0197 PMC8213005 33231474

[2] Kerchberger AM, Sheth AN, Angert CD, Mehta CC, Summers NA, Ofotokun I, Weight gain associated with integrase stand transfer inhibitor use in women. Clinical Infectious Diseases. 2020 7 27;71(3):593–600. 31504324 10.1093/cid/ciz853 PMC7384314

[3] Sax PE, Erlandson KM, Lake JE, Mccomsey GA, Orkin C, Esser S, Weight gain following initiation of antiretroviral therapy: risk factors in randomized comparative clinical trials. Clinical Infectious Diseases. 2020 9 15;71(6):1379–89. 31606734 10.1093/cid/ciz999 PMC7486849

[4] Bourgi K, Jenkins CA, Rebeiro PF, Shepherd BE, Palella F, Moore RD, Weight gain among treatment-naïve persons with HIV starting integrase inhibitors compared to non-nucleoside reverse transcriptase inhibitors or protease inhibitors in a large observational cohort in the United States and Canada. Journal of the International AIDS Society. 2020 4;23(4):e25484. 10.1002/jia2.25484 PMC7159248 32294337

[5] Debroy P, Sim M, Erlandson KM, Falutz J, Prado CM, Brown TT, Progressive increases in fat mass occur in adults living with HIV on antiretroviral therapy, but patterns differ by sex and anatomic depot. Journal of Antimicrobial Chemotherapy. 2019 4 1;74(4):1028–34. 10.1093/jac/dky551 PMC6419613 30668716

[6] Summers NA, Lahiri CD, Angert CD, Aldredge A, Mehta CC, Ofotokun I, Metabolic Changes Associated With the Use of Integrase Strand Transfer Inhibitors Among Virally Controlled Women. JAIDS Journal of Acquired Immune Deficiency Syndromes. 2020 11 1;85(3):355–62. 33060420 10.1097/QAI.0000000000002447 PMC7577246

[7] Kyle TK, Dhurandhar EJ, Allison DB. Regarding obesity as a disease: evolving policies and their implications. Endocrinology and Metabolism Clinics. 2016 9 1;45(3):511–20. 27519127 10.1016/j.ecl.2016.04.004 PMC4988332

[8] Blüher M Obesity: global epidemiology and pathogenesis. Nature Reviews Endocrinology. 2019 5;15(5):288–98. 10.1038/s41574-019-0176-8 30814686

[9] Gustafson DR, Mielke MM, Tien PC, Valcour V, Cohen M, Anastos K, Anthropometric measures and cognition in middle-aged HIV-infected and uninfected women. The Women’s Interagency HIV Study. Journal of Neurovirology. 2013 12;19(6):574–85. 24338243 10.1007/s13365-013-0219-1 PMC3957488

[10] Gustafson DR, Mielke MM, Keating SA, Holman S, Minkoff H, Crystal HA. Leptin, adiponectin and cognition in middle-aged HIV-infected and uninfected women. The Brooklyn women’s interagency HIV study. Journal of Gerontology & Geriatric Research. 2015 10;4(5). 10.4172/2167-7182.1000240 PMC4984413 27536467

[11] Arnoldussen IA, Gustafson DR, Leijsen EM, de Leeuw FE, Kiliaan AJ. Adiposity is related to cerebrovascular and brain volumetry outcomes in the RUN DMC study. Neurology. 2019 8 27;93(9):e864–78. 31363056 10.1212/WNL.0000000000008002

[12] Chan P, Brew BJ. HIV associated neurocognitive disorders in the modern antiviral treatment era: prevalence, characteristics, biomarkers, and effects of treatment. Current HIV/AIDS Reports. 2014 9 1;11(3):317–24. 24966139 10.1007/s11904-014-0221-0

[13] Alzheimer’s Association. 2021 Alzheimer’s Disease Facts and Figures. Alzheimer’s & Dementia. 2021;17(3):327–406. 10.1002/alz.12328 33756057

[14] Pedditizi E, Peters R, Beckett N. The risk of overweight/obesity in mid-life and late life for the development of dementia: a systematic review and meta-analysis of longitudinal studies. Age and Ageing. 2016 1 1;45(1):14–21. 26764391 10.1093/ageing/afv151

[15] Hassing LB, Dahl AK, Pedersen NL, Johansson B. Overweight in midlife is related to lower cognitive function 30 years later: a prospective study with longitudinal assessments. Dementia and Geriatric Cognitive Disorders. 2010;29(6):543–52. 20606436 10.1159/000314874 PMC3202952

[16] Livingston G, Sommerlad A, Orgeta V, Costafreda SG, Huntley J, Ames D, Dementia prevention, intervention, and care. The Lancet. 2017 12 16;390(10113):2673–734. 10.1016/S0140-6736(17)31363-6 28735855

[17] Mielke MM, Vemuri P, Rocca WA. Clinical epidemiology of Alzheimer’s disease: assessing sex and gender differences. Clinical Epidemiology. 2014;6:37. 24470773 10.2147/CLEP.S37929 PMC3891487

[18] Emmerzaal TL, Kiliaan AJ, Gustafson DR. 2003–2013: a decade of body mass index, Alzheimer’s disease, and dementia. Journal of Alzheimer’s Disease. 2015 1 1;43(3):739–55. 10.3233/JAD-141086 25147111

[19] Gustafson DR. Epidemiology informs randomized clinical trials of cognitive impairments and late-onset, sporadic dementias. Journal of Neurology & Neuromedicine. 2018;3(5):13–8. 33748680 10.29245/2572.942x/2018/5.1220 PMC7971422

[20] Franx BA, Arnoldussen IA, Kiliaan AJ, Gustafson DR. Weight loss in patients with dementia: considering the potential impact of pharmacotherapy. Drugs & Aging. 2017 6 1;34(6):425–36. 28478593 10.1007/s40266-017-0462-x PMC6779159

